# Supplementary material for: ChatGPT as a Tool for Biostatisticians: A Tutorial on Applications, Opportunities, and Limitations
Source: Stat Med. 2025 Oct 23;44(23-24):e70263. doi: 10.1002/sim.70263 (PMC12548020; doi:10.1002/sim.70263)
Supplement: Supplementary file 1 — Data S1. Supporting Information. [file SIM-44-0-s001.zip › Supplementary Materials Additional.zip!/3.7_Translation_Between_Multiple_Statistical_Programming_Languages/3.7.2_SPSS_to_R/3.7.2_Translation_Chat1.pdf]

OpenAI

R 1

Clear memory

I can't clear memory directly, but you can manage or clear your memory by following these steps:

1. Go to **Settings**.
2. Select **Personalization**.
3. Click on **Memory**.
4. From there, you can view what I remember and clear specific items or everything.

Let me know if you want help navigating there or understanding what's stored!

Take the role of an expert biostatistician for this chat

Got it — stepping into expert biostatistician mode now. Feel free to throw any data, study designs, statistical models, or analysis questions my way. Whether it's clinical trials, survival analysis, mixed models, or anything else biostats-related, I'm ready. What's on your mind?

I am running the following analysis in SPSS:
DATASET ACTIVATE DataSet5. LOGISTIC REGRESSION VARIABLES V2 /METHOD=FSTEP(LR) V3 V4 V5 V6 V7 V8 V9 V10 V11 V12 V13 V14 V15 V16 V17 V18 V19 V20 V21 V22 V23 V24 V25 V26 V27 V28 V29 V30 V31 V32 /CRITERIA=PIN(0.05) POUT(0.10) ITERATE(20) CUT(0.5).
Translate this into R code.
The data is loaded into R with
temp <- tempfile()
download.file(
 "https://archive.ics.uci.edu/static/public/17/breast+cancer+wisconsin+diagnostic.zip",
 temp
)
data <- read.table(unz(temp, "wdbc.data"), sep = ",")
data$V2 <- data$V2 == "M"
unlink(temp)

Great — you're running a **forward stepwise logistic regression using likelihood ratio tests** in SPSS. Let's translate that into R.

You're using:

- `V2` as the **binary outcome** (which you've already converted to a logical `TRUE`/`FALSE` format).

- Predictors: `V3` to `V32`.
- A **forward stepwise (likelihood ratio)** method.
- Criteria: include variables with p-value < 0.05 (`PIN`), remove if p > 0.10 (`POUT`).
- 20 iterations max.

R Equivalent (Using `MASS::stepAIC`)

R doesn't natively do LR-based stepwise regression with PIN/POUT directly. But a widely accepted approach is using **AIC-based stepwise selection** via the `stepAIC()` function from the `MASS` package. This mimics forward stepwise reasonably well.

Here's how to replicate your analysis in R:

```r
# Load necessary libraries
library(MASS)

# Set outcome variable as factor for logistic regression
data$V2 <- factor(data$V2, levels = c(FALSE, TRUE))  # Ensure proper binary coding

# Fit null model (intercept only)
null_model <- glm(V2 ~ 1, data = data, family = binomial)

# Fit full model with all predictors V3 to V32
full_model <- glm(V2 ~ ., data = data[, -1], family = binomial)  # exclude V1 (ID column)

# Perform forward stepwise selection
stepwise_model <- stepAIC(null_model,
                          scope = list(lower = null_model, upper = full_model),
                          direction = "forward",
                          trace = TRUE)
```

Notes:

- `V1` seems like an ID column — excluded from modeling.
- `stepAIC` uses **AIC** rather than p-values, but it's commonly used in biostats for model selection.
- R does **not** use `PIN/POUT` by default, but if you want p-value-driven selection, we can code a custom procedure using `add1()` and `drop1()`.

Would you like the **custom p-value-based forward selection** in R instead of AIC-based?

Yes

Perfect — let's implement **custom forward stepwise logistic regression in R based on p-values**, like SPSS does using likelihood ratio tests.

🧠 Goal:

We want to:

- Start with a **null model**.
- At each step, **add the variable with the smallest p-value** (from LRT) if p < 0.05.
- Once added, check all variables in the model and **drop any with p > 0.10**.
- Repeat until no variables meet the add/drop criteria.
- Logistic regression model (binomial family).

✅ Here's the code to do this:

```r
# Set up: outcome is V2 (TRUE = malignant, FALSE = benign)
data$V2 <- factor(data$V2, levels = c(FALSE, TRUE))

# List of predictor variable names (V3 to V32)
predictors <- paste0("V", 3:32)

# Start with null model
current_formula <- as.formula("V2 ~ 1")
current_model <- glm(current_formula, data = data, family = binomial)

# Track included predictors
included <- c()
remaining <- predictors
changed <- TRUE

while (changed) {
  changed <- FALSE

  # === Try adding one variable at a time ===
  add_candidates <- list()

  for (var in remaining) {
    test_formula <- as.formula(paste("V2 ~", paste(c(included, var), collapse = " + ")))
    test_model <- glm(test_formula, data = data, family = binomial)
    lrt_p <- anova(current_model, test_model, test = "LRT")[2, "Pr(>Chi)"]
    add_candidates[[var]] <- lrt_p
  }

  # Find best variable to add
  best_add <- names(which.min(unlist(add_candidates)))
  best_add_p <- add_candidates[[best_add]]

  if (!is.null(best_add) && best_add_p < 0.05) {
    included <- c(included, best_add)
    remaining <- setdiff(remaining, best_add)
    current_formula <- as.formula(paste("V2 ~", paste(included, collapse = " + ")))
    current_model <- glm(current_formula, data = data, family = binomial)
    changed <- TRUE
  }

  # === Try removing variables that no longer meet POUT criteria ===
  if (length(included) > 1) {
    drop_test <- drop1(current_model, test = "LRT")
    drop_p <- drop_test[-1, "Pr(>Chi)"]

    remove_vars <- names(drop_p[drop_p > 0.10])

    if (length(remove_vars) > 0) {
      included <- setdiff(included, remove_vars)
      remaining <- c(remaining, remove_vars)
      current_formula <- as.formula(paste("V2 ~", paste(included, collapse = " + ")))
      current_model <- glm(current_formula, data = data, family = binomial)
      changed <- TRUE
    }
  }
}
```

```
summary(current_model)
```

🔍 What this does:

- Emulates **SPSS forward stepwise LR** using p-values (PIN = 0.05, POUT = 0.10).
- Starts with null model and evaluates each variable for entry based on **likelihood ratio p-value**.
- Removes variables if their LRT p > 0.10 after being added.
- Stops when no more variables qualify to enter or exit.

Let me know if you want:

- A table of step-by-step model changes
- ROC curve or accuracy evaluation
- Export of final variable list or coefficients

Happy to build on it!
